# Supplementary material for: Rational Food Design Targeting Micronutrient Deficiencies in Adolescents: Nutritional, Acoustic-Mechanical and Sensory Properties of Chickpea-Rice Biscuits
Source: Foods. 2023 Feb 23;12(5):952. doi: 10.3390/foods12050952 (PMC10000554; doi:10.3390/foods12050952)
Supplement: Supplementary file 1 [file foods-12-00952-s001.zip › foods-2212345-supplementary.pdf]

MEMBER OF BASQUE RESEARCH & TECHNOLOGY ALLIANCE

PRODUCT TASTING WITH SCHOLARS (11-12 y). CHICKPEA BISCUITS

For a correct performance of the test, please read carefully the instructions that will appear throughout the session. If in doubt, consult the staff in the room.

1. Enter your email address.

THE PRODUCT:

In today's tasting we are going to try a type of plain biscuit.

SENSORY EVALUATION OF THE PRODUCT:

2. Please taste the sample and indicate how much the following characteristics of the product were liked with the help of the scale:

*The consumers were asked to record their liking intensity scores for appearance, colour, aroma, flavour and texture. A 9-point hedonic scale was used (9 = like extremely and 1 = dislike extremely).

A print-screen of the questionnaire is shown in Spanish.

	Me disgusta muchísimo	Me disgusta mucho	Me disgusta bastante	Me disgusta ligeramente	Ni me gusta ni me disgusta	Me gusta ligeramente	Me gusta bastante	Me gusta mucho	Me gusta muchísimo
	1	2	3	4	5	6	7	8	9
Apariencia	❍	❍	❍	❍	❍	❍	❍	❍	❍
Color	❍	❍	❍	❍	❍	❍	❍	❍	❍
Olor	❍	❍	❍	❍	❍	❍	❍	❍	❍
Sabor	❍	❍	❍	❍	❍	❍	❍	❍	❍
Textura	❍	❍	❍	❍	❍	❍	❍	❍	❍
Comentarios:									

3. In your opinion, the option that best describes the overall quality of this snack is:

	Me disgusta muchísimo	Me disgusta mucho	Me disgusta bastante	Me disgusta ligeramente	Ni me gusta ni me disgusta	Me gusta ligeramente	Me gusta bastante	Me gusta mucho	Me gusta muchísimo
	1	2	3	4	5	6	7	8	9
	❍	❍	❍	❍	❍	❍	❍	❍	❍
Comentarios:									

4. Are you able to identify a particular flavour? Please specify

PRODUCT TASTING WITH SCHOLARS (11-12 y). CHICKPEA BISCUITS

CONSUMPTION HABITS:

5. Do you consume biscuits?

a) Yes (next question)

b) No (skip next question)

6. How often do you eat biscuits?

1. twice a week or more often
2. once a week
3. twice a month
4. once a month
5. once every 2–3 months
6. less frequently

SOCIODEMOGRAPHIC DATA:

The data that you are going to fill in below will be used only for the preparation of this work and will be treated confidentially.

7. Year of birth:

8. Gender:

a) Female

b) Male

c) Non-binary

END OF SENSORY EVALUATION SESSION:

You have reached the end of this session.

Thank you very much and see you soon.

Social and Consumer Science Laboratory Team
